# Risks and management of hypertension in cancer patients undergoing targeted therapy: a review

**DOI:** 10.1186/s40885-022-00197-3

**Published:** 2022-05-15

**Authors:** Xiaolei Zhu, Shenhong Wu

**Affiliations:** 1 Division of Primary Care, Department of Medicine, Renaissance School of Medicine at Stony Brook University, 205 North Belle Mead Road, NY 11733 Stony Brook, USA; 2 Division of Hematology and Oncology, Department of Medicine, Renaissance School of Medicine at Stony Brook University, Lauterbur drive, NY 11794 Stony Brook, USA

**Keywords:** Hypertension, Molecular targeted therapy, Risk, Mechanism of action, Diagnosis, Disease management

## Abstract

**Background:**

Rapid progress over the last decade has added numerous agents targeting specific cellular signaling pathways to the treatment armamentarium for advanced cancer. However, many of these agents can cause hypertension resulting in major adverse cardiovascular event.

**Methods and results:**

A systematic literature search was performed on the databases PubMed and Google Scholar for papers published in English until December 2020. This review summarizes the risk, mechanism, diagnosis, and management of hypertension in cancer patients undergoing targeted therapy. The risk and pathogenesis of hypertension vary widely with different classes of targeted agents. Currently there is a paucity of data investigating optimal management of hypertension with targeted therapy. A practical approach is discussed with a focus on the goal of blood pressure control as well as drug selection based on the mechanism of hypertension in the context of advanced cancer, treatment toxicity, comorbidity, and drug-drug interactions. This review also discusses many studies that have explored hypertension as a biomarker for cancer treatment efficacy and as a pharmacodynamic biomarker to titrate drug dose.

**Conclusions:**

The diversity of targeted agents has provided important insights into the pathogenesis of hypertension in cancer patients. The underlying mechanism may provide a guidance to the management of hypertension. Further studies are needed to investigate optimal treatment and hypertension as a biomarker for cancer treatment.

## Background

Great stride has been made on cancer treatment leading to improved survival in recent years, particularly with clinical application of numerous drugs designed to specifically target specific signaling pathways of malignant cells. Even though cancer incidence has been relatively stable, the mortality trend has slowed significantly [1]. Malignancies that were previously uncontrollable can now be turned into chronic diseases, with many patients on targeted therapeutic agents to control progression for long term. However, targeted therapy is associated with significant side effects such as hypertension and cardiovascular risks.

Hypertension is the most common cardiovascular morbidity in cancer registries with a prevalence of 37% [2]. Many cancer patients have common risk factors for primary hypertension such as advanced age, obesity, and diabetes; they also have many risk factors for secondary hypertension such as pain, anxiety, renal dysfunction, malignancy and its treatment. A variety of cancer treatment could cause hypertension, including targeted therapy, alkylating agents, radiation therapy that causes renal artery stenosis, baroreflex failure, nephrectomy/renal disease, steroid, and erythropoietin use. Targeted agents have emerged as a major risk factor for secondary hypertension, leading to serious cardiovascular events. Over the last decade, it has been well established that hypertension is commonly caused by agents interfering with vascular endothelial growth factor (VEGF) pathways [3, 4]. In addition, hypertension is a common risk for those agents affecting androgen-signaling such as abiraterone and enzalutamide [5, 6]. Other agents frequently associated with hypertension include proteasome inhibitors (PIs) such as carfilzomib [7], phosphatidylinositol 3-kinase inhibitor such as copanlisib [8], and Bruton tyrosine kinase inhibitor (TKI) such as ibrutinib [9]. Interestingly, hypertension can be a biomarker indicative of good outcome, and has been used to titrate the optimal dose of targeted therapy in some instances [10, 11]. On the other hand, blood pressure (BP) can be labile for many cancer patients due to poor appetite, diarrhea, weight loss associated with treatment toxicity and disease status, resulting a difficulty in appropriate control with antihypertensive medications. The complexity of hypertension in this setting has presented as a major challenge for medical professionals. With the extensive and long-standing use of these agents, it is becoming a part of routine problem list for internists as well as oncologists. Currently, the optimal approach to the management of hypertension associated with targeted therapy has not been established.

In this review, we summarize recent studies on the risks and pathogenesis of hypertension associated with targeted agents. We also discuss a practical approach to individualized care focusing on the goal of BP control with close monitoring and antihypertensive drug selection based on etiology and mechanisms. Finally, we summarize recent data on hypertension as a biomarker for the targeted therapy in cancer patients.

## Methods

A systematic literature review was performed on the databases PubMed and Google Scholar for papers published in English between 1 and 2005, and 30 December 2020. We used keywords such as “targeted therapy,” “angiogenesis inhibitors,” “bevacizumab,” “sorafenib,” “sunitinib,” “abiraterone,” “enzalutamide,” “ibrutinib,” “carfilzomib,” “Copanlisib,” “hypertension,” and “cancer”. Clinical trials and their meta-analysis were included. We also searched the database (clinicaltrials.gov) for relevant clinical trials regarding hypertension and cancer. In addition, observational clinical studies, such as cohort, case-control and cross-sectional studies, were included. Reviews and editorials were included when deemed relevant and related to the topic.

### Risk and pathogenesis of hypertension with targeted therapy

**Fig. 1:**
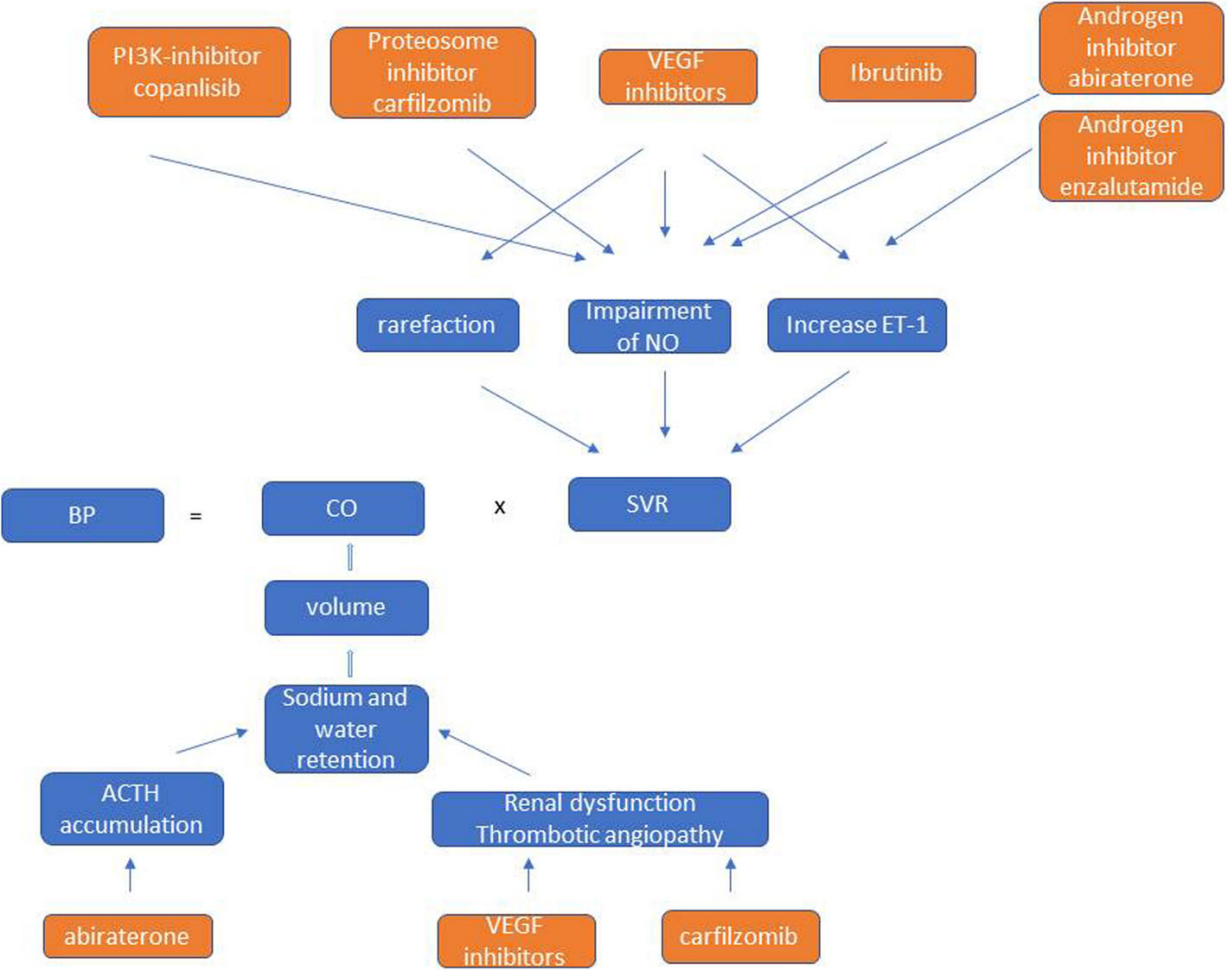
Mechanism of hypertension secondary to targeted therapy. Different targeted anti-cancer agents (brown color) can have a variety of distinct effects on the development of hypertension due to increased systematic vascular resistance and cardiac output. VEGF, vascular endothelial growth factor; NO, nitric oxide; ET-1, endothelin-1; BP, blood pressure; CO, cardiac output; SVR, systematic resistance; ACTH, adrenal cortical trophic hormone

Several categories of targeted therapeutic agents have been associated with the increased risk of hypertension based mostly on clinical trial data including randomized trials, meta-analyses, and single-arm studies, with VEGF inhibitors being the most extensively studied. We focused on incidences and relative risks (RR) when results were available (Table 1) [12–22]. The pathogenesis of hypertension varied widely among these agents affecting systematic vascular resistance or sodium/water retention, and is summarized in Fig. 1.

**Table 1:** Incidence and RR of all-grade and high-grade hypertension with targeted therapies

Type of cancer therapy		All-grade hypertension		High-grade hypertension		Reference
		Incidence (95% CI), %	RR (95% CI)	Incidence (95% CI), %	RR (95% CI)	
Monoclonal antibodies targeting VEGF	Bevacizumab	23.6 (20.5–27.1)	3.02 (2.24–4.07)	7.9% (6.1–10.2%)	5.28 (4.15–6.71)	[12]
	Ramucirumab	16.4 (11.9–22.3)	2.28 (1.61–3.24)	9.8 (7.2–13.0)	3.59 (2.32–5.53)	[13]
VEGF fusion molecules	Aflibercept	42.4 (35.0–50.3)	4.47 (3.84–5.22)	17.4 (13.7–21.9)	4.97 (3.95–6.27)	[14]
Tyrosine kinase inhibitors	Sorafenib	19.1 (15.8–22.4)	3.07 (2.05–4.60)	4.3 (3.0–5.5)	3.31 (2.21–4.95)	[15]
	Sunitinib	21.6 (18.7–24.8)	3.44 (0.62–19.15)	6.8 (5.3–8.8)	22.72 (4.48–115.29)	[16]
	Pazopanib	35.9 (31.5–40.6)	4.97 (3.38–7.30)	6.5 (5.2–8.0)	2.87 (1.16–7.12)	[17]
	Axitinib	40.1 (30.9–50.2)	3.0 (1.29–6.97)	13.1 (6.7–24)	1.71 (1.21–2.43)	[18]
	Regorafenib	44.4 (30.8–59.0)	3.76 (2.35–5.99)	12.5 (5.2–27.1)	8.39 (3.10–22.71)	[19]
	Cabozantinib	39.0 (33.9–44.3)	5.48 (3.76–7.99)	17.0 (12.4–20.4)	5.09 (2.71–9.54)	Incidence: PI 2020^a)^ RR: [20]
	Vandetanib	24.2 (18.1–30.2)	5.1 (3.76–6.92)	6.4 (3.3–9.5)	8.06 (3.41–19.04)	[21]
	Lenvatinib	47.0 (35.4–58.9)	3.47 (2.31–5.21)	17.7 (10.2–28.9)	11.54 (4.83–27.57)	Incidence: [22] RR: PI 2020
	Ponatinib	45.5 (36.7–54.5)	NA	22.7 (14.5–33.8)	NA	Incidence: PI 2020
	Nilotinib	10.0 (7.0–14.2)	4.01 (1.78–9.04)	1.1 (0.3–3.3)	3.01 (0.32–28.77)	Incidence: PI 2020 RR: compared with imatinib
Androgen inhibitors	Abiraterone	21.9 (13.6–33.2)	1.80 (1.47–2.19)	10.2 (6.9–11.6)	2.11 (1.66–2.68)	[5]
	Enzalutamide	11.9 (8.8–16.0)	2.82 (2.34–3.38)	4.9 (3.5–6.8)	2.27 (1.73–2.96)	[6]
Proteasome inhibitors	Bortezomib	13 (10.0–17.3)	1.78 (1.12–2.84)	2.6 (1.4–5.0)	4.46 (0.97–20.49)	PI 2020
	Carfilzomib	12.2 (9.8–14.9)	2.71 (1.53–4.82)	4.3 (2.6–6.4)	3.0 (0.97–9.15)	Incidence: [7] RR: PI 2020
PI3 kinase inhibitors	Copanlisib	35.0 (28.3–42.6)	NA	26 (21.3–31.0)	NA	Incidence: PI 2020
Bruton tyrosine kinase inhibitor	Ibrutinib	19 (17.1–21.1)	2.82 (1.52–5.23)	8 (6.7–9.5)	RR: NA	Incidence: PI 2020. RR: [9]

The data were derived from systematic review and meta-analysis or calculated from the package inserts if not available as indicated

RR, relative risk; CI, confidence interval; VEGF, vascular endothelial growth factor; PI, proteasome inhibitor; NA, no available data for calculations

^a)^Calculated based on PI 2020

### Angiogenesis inhibitors

Drugs blocking VEGF pathway extensively used for the treatment of multiple metastatic cancers (malignancy of lung, stomach, colon, liver, thyroid, and kidney; and certain sarcomas) include recombinant humanized monoclonal antibodies binding to VEGF-A such as bevacizumab, and the extracellular domain of VEGF receptor (VEGFR) such as ramucirumab, a soluble decoy receptor such as aflibercept, and TKIs that act intracellularly on the tyrosine kinase domain of VEGFR such as lenvatinib and cabozantinib (Table 1). Almost all the clinical trials have shown that VEGF inhibitors caused an increase in BP, leading to all-grade and high-grade hypertension [3, 4, 23]. The risk of hypertension with VEGF inhibitors was substantial but varied widely with different drugs (Table 1). The highest incidence of all-grade hypertension was observed with lenvatinib (47.0%; 95% confidence interval [CI], 35.4–58.9%), and the lowest was seen with ramucirumab (16.4%; 95% CI, 11.9–22.3%); in comparison with controls, the highest RR was observed with cabozantinib (5.48; 95% CI, 3.76 to 7.99), and the lowest RR was seen with ramucirumab (2.28; 95% CI, 1.61 to 3.24). Pre-existing hypertension, age >60 years, and body mass index >25 kg/m^2^ could help to identify patients at risk for significant anti-VEGF therapy-induced BP elevation [24]. Notably, non-VEGFR TKI nilotinib with anti-angiogenesis properties (targeting BCR-ABL, c-kit, platelet-derived growth factor) also significantly increased the risk of hypertension with an RR of 4.01 (95% CI, 1.78 to 9.04).

Major mechanisms for this type of hypertension may include the decrease of VEGF signaling leads to reduced production of vasodilators such as nitric oxide (NO) and prostaglandin 2 (PGI2, also called prostacyclin) [4, 25], increased production of vasoconstrictors such as endothelin-1, and increased vascular tone and arterial remodeling with oxidative stress and rarefaction [26, 27]. In addition, renal dysfunction such as thrombotic angiopathy can contribute to the development of hypertension. Similar pathogenesis may be present in eclampsia, with the identification of placental secreted antiangiogenic factors (soluble fms-like tyrosine kinase-1 and endoglin) [28].

### Androgen signaling inhibitors

The incidences of all-grade and high-grade hypertension for abiraterone were 21.9% (95% CI, 13.6–33.2%) and 10.2% (95% CI, 6.9–11.6%), respectively; and the incidences of all-grade and high-grade hypertension for enzalutamide were 11.9% (95% CI, 8.8–16.0%) and 4.9% (95% CI, 3.5–6.8%), respectively. Both abiraterone and enzalutamide are associated with similarly increased risk of all-grade hypertension with RRs of 1.80 (95% CI, 1.47 to 2.19) and 2.81 (95% CI, 2.34 to 3.38), respectively; and high-grade hypertension with RRs of 2.11 (95% CI, 1.66 to 2.68) and 2.27 (95% CI, 1.73 to 2.96), respectively [5, 6]. In addition, both drugs increased cardiovascular risk significantly with RR of 1.98 for all-grade and 2.26 for high-grade [29].

Abiraterone as a selective inhibitor of androgen biosynthesis that potently blocks cytochrome P450 (cyp17) causes concurrent suppression of cortisol leading to increase of adrenal cortical trophic hormone which in turn causes mineral corticoid excess. Abiraterone may cause hypertension due to increased mineral corticoid production, reduced androgen synthesis with metabolic syndrome, and anti-cancer effect. Androgen deficiency was associated with vasoconstriction and smooth muscle proliferation via endothelia dysfunction [30]. Anti-tumor effect may contribute to the increase of BP due to decreased VEGF secretion, weight gain and improved appetite. Enzalutamide targets androgen receptor and its signaling pathway by competitively binding to the ligand-binding domain of the androgen receptor [31], and maybe similar to abiraterone in causing hypertension through androgen deficiency and anti-tumor effect [30]; In addition, it may affect androgen-related vasodilatation with L-type calcium channel in small arteries independent of the endothelium [32].

### Proteasome inhibitors

The incidences of all-grade and high-grade hypertension with carfilzomib, which is frequently used to treat multiple myeloma, were 12.2% (95% CI, 9.8–14.9%) and 4.3% (95% CI, 2.6–6.4%) [7, 33]. Addition of carfilzomib to chemotherapy significantly increased the risk of all-grade hypertension with an RR of 2.71 (95% CI, 1.53 to 4.82) in comparison with controls based on the calculation from package insert. It may also increase the risk of high-grade hypertension including hypertensive crisis. In the ENDEAVOR trial, the incidence of grade 3 or higher hypertension was 15% in the carfilzomib arm and 3% in the bortezomib arm [34, 35]. Interestingly, another PI bortezomib is associated with orthostatic hypotension (bortezomib product characteristics) [36]. The difference in BP effect between the two agents may be explained by carfilzomib being a much stronger and irreversible PI [37]. They may affect BP by reducing NO through proteasome-mediated endothelial nitric oxide synthase (eNOS) regulation [38]. In addition, thrombotic microscopic angiopathy was reported in carfilzomib-related hypertension [39].

### PI3K inhibitor

The elevation of BP with the PI3K inhibitor copanlisib was frequently observed during its infusion [40]. In the clinical trial involving lymphoma patients, about 30% patients developed hypertension or worsening BP, with 20% patients having grade 3 or higher [8]. Based on its package insert, the overall incidences of all-grade and high-grade hypertension were calculated to be 35.0% (95% CI, 28.3–42.6%) and 26.0% (95% CI, 21.3–31.0%), respectively. The mechanism of hypertension is not clear. One possible explanation may be involving PI3K/AKT/eNOS-dependent pathway [41].

### B-cell receptor signaling inhibitors

Based on the calculation from the package insert, the incidences of all-grade and high-grade hypertension with ibrutinib were 19.0% (95% CI, 17.1–21.1%) and 8.0% (95% CI, 6.7–9.5%), respectively. The incidence of hypertension was also high in recent data from The Ohio State University’s Comprehensive Cancer Center with 38% being high-grade, and more than 75% of patients developed new or worsened hypertension during therapy [42]. In some clinical trials, ibrutinib was associated with nearly threefold increase in the incidence of high-grade hypertension, and the incidence of overall hypertension was 44% [43]. Meta-analysis from eight randomized controlled trials showed that ibrutinib was associated with a significant increase in the risk of hypertension with an RR of 2.82 (95% CI, 1.52 to 5.23) [9]. Ibrutinib may cause hypertension by inhibiting PI3K/AKT pathway, including indirect down-regulation of PI3K-p110α [44, 45], and down-regulating VEGF [46] resulting in NO reduction and endothelium dysfunction in a fashion similar to VEGF inhibitors [47].

### Diagnosis and general management of hypertension with targeted therapy

Diagnosis of hypertension in cancer patients is similar to that of non-cancer patients. The diagnosis of hypertension is based appropriately measured BP ≥130/80 mmHg suggested by 2017 American College of Cardiology/American Heart Association (ACC/AHA) guideline. BP should be checked in both arms unless there are contraindications including indwelling catheters and lymphoedema. It should be checked before, during, and after the course of treatment. Closely monitoring BP at home is especially important. Evidence suggests that white coat hypertension and masked hypertension may be more common in individuals receiving cancer treatment compared with general population [48, 49]. In addition, home BP is a better predictor than office BP readings. Its monitoring is more significant when targeted therapy is administered with on-off schedules since BP might drop during the off-period. It is highly recommended to utilize ambulatory BP monitoring over a 24-hour period as a means of diagnosis rather than spot test. Diagnosis via ambulatory monitoring is the gold standard due to a stronger association with cardiovascular outcomes, reflecting the hypertension ‘load’ over the 24 h. The diagnostic threshold in ambulatory monitoring is often lowered to an average BP ≥125/75 per 2017 ACC/AHA guideline. Telehealth monitoring of BP has demonstrated utility. An ongoing trial is to develop a process using a wireless BP monitor and automated uploading/messaging system with their primary care provider and oncologist for the improvement of BP management in cancer patients (NCT03919214).

After the initial diagnosis of hypertension, the next essential step is to consider all the etiologies and contributing factors in order to differentiate between primary and secondary hypertension, including pain issues that are common in cancer patients receiving targeted therapy due to metastatic lesions or procedures, any general supportive treatment such as intravenous fluids with high sodium content, and anxiety related BP elevation. And finally, the temporal relationship of hypertension with treatment is important to assess cause-effect with newly-onset or worsening hypertension after targeted therapy being highly indicative of its direct association.

There is a paucity of randomized controlled studies regarding the optimal management of hypertension in cancer patients receiving targeted therapy. We propose a practical approach to individualized management with a focus on the goal of BP control and drug selection based on etiology and mechanism. Currently, 2017 ACC/AHA guideline recommend goal of BP control with medication is <130/80 mmHg. However, individualized approach should be considered in certain groups. For elderly patients >80 years of age, systolic BP of 140–150 mmHg might be more appropriate [50]; for patients who have orthostatic hypotension, we might have to accept slightly higher BP goal to avoid falls. The approach also requires to manage hypertension according to underlying etiologies. For patients with primary hypertension or in the situation where it was difficulty to dissect the contribution of various factors, we should follow general guidelines for BP control. For patients with hypertension secondary to specific causes other than targeted therapy, it is important to address those contributing factors.

### Management of hypertension with targeted therapy: special considerations

A practical approach is to select specific antihypertensive medications according to different mechanisms of action, pathogenesis, and drug-drug interactions due to the paucity of strong evidence for optimal treatment at this time. Special considerations in the context of targeted therapy have been summarized in Table 2. In the setting of poorly-controlled hypertension, dose reductions and/or interruptions of targeted therapy should be started.

**Table 2:** Special considerations for the management of hypertension in patients undergoing targeted therapy

Targeted therapy drug class	Preferred choice	Might be helpful	Drugs need to avoid
VEGF inhibitors - VEGF antibody - TKI	ACEI/ARB Dihydropyridine calcium channel blocker	Nitrates	Avoid non-dihydropyridine calcium channel blocker for TKI users
Androgen inhibitors - Abiraterone - Enzalutamide	Eplerenone for abiraterone	Dihydropyridine calcium channel blocker	Avoid spironolactone for abiraterone
Proteasome inhibitors (carlifilzomib)	ACEI/ARB Beta-blocker	-	-
PI3K inhibitor	-	ACEI/ARB	-
Oral Bruton’s tyrosine kinase inhibitor	Beta-blocker	-	Avoid non-dihydropyridine calcium channel blocker. Avoid medications that potentially can increase heart rate.

VEGF, vascular endothelial growth factor; TKI, tyrosine kinase inhibitor; ACEI, angiotensin-converting enzyme inhibitor; ARB, angiotensin II receptor blocker

### Hypertension with vascular endothelial growth factor signaling inhibitors

The first-line treatment of hypertension in general population has been angiotensin-converting enzyme inhibitor (ACEI)/angiotensin receptor blocker (ARB), calcium channel blocker or thiazide diuretics. There are early evidences that suggest ACEI/ARB is the drug of choice especially in certain urinary cancers. ACEI/ARB was postulated to be preferred antihypertensive medication due to survival benefit in certain cancers such as renal cell carcinoma [51]. ACEI/ARB use was significantly associated with better overall survival (OS; hazard ratio [HR], 0.40; 95% CI, 0.24 to 0.66; *P* < 0.001) and progression-free survival (PFS; HR, 0.55; 95% CI, 0.35 to 0.86; *P* = 0.009) in metastatic renal cell cancer patients with VEGF therapy [52]. A meta-analysis comparing the use and non-use of ACEIs or ARBs in several types of cancer (4,964 patients treated in a total of 11 trials) showed that the use of ACEIs or ARBs resulted in a significant improvement in disease-free survival (HR, 0.60; 95% CI, 0.41 to 0.87; *P* = 0.007) and OS (HR, 0.75; 95% CI, 0.57 to 0.99; *P* = 0.04) [53]. It was also preferred due to its anti-proteinuria effect since VEGF inhibition is related to the high incidence of proteinuria in addition to hypertension [54]. Thus, ACEI/ARB can be considered as first-line treatment if there is no contraindication such us hyperkalemia, advanced kidney disease or severe bilateral renal artery stenosis that ACEI/ARB can’t be tolerated.

Dihydropyridine calcium channel blocker such as amlodipine is effective due to its vasodilatory mechanism. Amlodipine 5 mg daily appears safe and efficient for the treatment of hypertension in patients receiving bevacizumab [55]. Non-dihydropyridine calcium channel blockers such as verapamil and diltiazem which are strong CYP3A4 inhibitors can cause drug-drug interactions with many TKIs that are metabolized through cytochrome P450. The use of thiazide diuretics in patients receiving anti-VEGF therapy needs precaution due to diarrhea frequently associated with TKIs leading to hypovolemia and hypokalemia. Due to inhibition of NO production in VEGF therapy, nitrate therapy is an attractive option.

### Hypertension with androgen signaling inhibitors

Due to the side effect of hypokalemia and fluid retention as a consequence of mineralocorticoid excess resulting from CYP17 inhibition with abiraterone, eplerenone is a drug of choice due to its diuretic effect and the ability to reduce potassium wasting [56]. However, spironolactone, another potassium sparing diuretic, is not recommended due to its potential side effect of promoting prostate tumor growth by compromising the therapeutic effect of abiraterone [57]. Mechanistically, it is reasonable to treat enzalutamide associated hypertension as a part of metabolic syndrome secondary to hypogonadism using diet, exercise and standard antihypertensive medications. Calcium channel blocker might be highly effective in the setting due to the enzalutamide effect on L-type calcium channel.

### Hypertension with proteasome inhibitors

Given the link between carfilzomib and NO hemostasis, medications that targeting at NO releasing agent such as nitrates might be of interest. In addition, heart failure incidence was noticed to be higher in carfilzomib group compared to control group [58], with hypertension often precedes left ventricular diastolic and systolic dysfunction [59]. Since ACE inhibitors, eplerenone, and β-blocker are the recommended first-line treatment for heart failure with reduced ejection fraction (EF< 35%), they may be considered as preferred choices.

### Ibrutinib-related hypertension

No specific single antihypertensive medication seems to be more effective than others [42]. Dickerson et al. [42] showed that the treatment of hypertension is associated with subsequent lower risk of major adverse cardiovascular event (MACE. HR, 0.40; 95% CI, 0.24 to 0.66). Coadministration of ibrutinib-a sensitive CYP3A4 substrate with a moderate to strong CYP3A4 inhibitor should be avoided due to the resulting increase of ibrutinib concentration. Due to the high risk of atrial fibrillation with ibrutinib, β-blocker might be a potential candidate. Medications that cause tachycardia such as hydralazine should be used with caution.

### Hypertension with PI3K inhibitors

CE-I/ARB may be particularly effective in PI3K-induced hypertension, because angiotensin II has been shown to require PI3K [60]. Copanlisib is mainly metabolized via CYP3A, thus medications that inhibit CYP3A4 such as diltiazem should be used with caution or avoided since it might increase copanlisib AUC (area under concentration).

### Hypertension as a biomarker for cancer treatment

BP elevation is considered as a class-effect of many targeted therapeutic agents, particularly angiogenesis inhibitors, and may reflect their biological effect on tumors. Several studies have investigated hypertension as a potential prognostic biomarker for treatment efficacy. In an observational study of 119 patients with advanced non-small cell lung cancer, colorectal cancer, or ovarian cancer receiving bevacizumab and chemotherapy, very early hypertension (within 42 days) was predictive of responses (*P* = 0.0011) using home-based measurements twice daily [61]. In a retrospective study of 39 colorectal cancer patients receiving bevacizumab as first-line therapy in combination with chemotherapy, hypertension is associated with better median progression-free survival (PFS: 14.5 mo vs. 3.1 mo; *P* = 0.04) [62]. In a total of 101 consecutive patients with metastatic colorectal cancer who were treated with standard chemotherapy combined with bevacizumab, hypertension was associated with improved PFS (10.5 mo vs. 5.3 mo; *P* = 0.008) and overall survival (OS: 25.8 mo vs. 11.7 mo; *P* < 0.001) [63]. A similar study in 181 colorectal cancer patients also showed that hypertension was associated with significantly better survival (*P* = 0.029) and better PFS (*P* = 0.024) [64]. In a pooled analysis from four perspective clinical trials of gemcitabine-based therapy combined with bevacizumab for advanced pancreatic cancer, hypertension is associated with significantly improved median OS, time to tumor progression and disease control rate [65]. In a retrospective study of renal cell cancer treated with sunitinib involving 544 patients from four clinical trials, PFS (HR, 0.60; 95% CI, 0.45 to 0.81) and OS (HR, 0.33; 95% CI, 0.25 to 0.43) were significantly improved in patients with treatment-induced hypertension [10]. Similar results were observed for sunitinib in 319 patients with gastrointestinal stromal tumor [66]. In a pooled study of five phase II studies of single agent axitinib for the treatment of four different tumor types, diastolic BP is correlated with clinical outcome including objective response rate, PFS, and OS [11]. However, hypertension was not associated with better PFS and OS in a retrospective study of 337 patients for advanced non-adipocytic soft tissue sarcoma treated with pazopanib [67], reflecting the complexity of correlation due to a difference in tumor types and TKI.

Hypertension has also been examined as a pharmacodynamic biomarker for the dose titration of axitinib in patients with metastatic renal cell carcinoma [68]. In the randomized, double-blind phase 2 study, 213 patients received axitinib 5 mg twice daily during a 4-week lead-in period, of whom 112 patients with BP 150/90 mmHg or lower, no grade 3 or 4 treatment-related toxic effects, no dose reductions, and no more than two antihypertensive drugs for two consecutive weeks were randomly assigned to the axitinib titration group or the placebo titration group. The greater proportion of patients in the axitinib titration group achieving an objective response than the placebo group (54% vs. 34%), supporting the concept of individual dose titration of axitinib. In the package insert, a dose increase is recommended for patients who tolerate axitinib for at least two consecutive weeks with no adverse reactions grade >2, are normotensive, and are not receiving anti-hypertension medications.

## Conclusions

The risk of hypertension varied significantly among different targeted agents with highest observed in patients receiving anti-VEGF agents. The pathogenesis of hypertension in these patients differed widely with various signaling pathways affected. In the absence of strong evidence for optimal management, a practical approach would focus on the goal of BP control based on the condition of individual cancer patient with close monitoring and drug selection based on etiology and mechanism of hypertension at this time. Further multi-institutional prospective studies are needed to investigate treatment according to the class of targeted agents due to the diversity of targeted therapy. Hypertension has also been explored as a biomarker for targeted therapy largely based on retrospective studies, further prospective studies are needed to confirm hypertension or BP elevation as a biomarker for cancer treatment efficacy.

## Data Availability

It will be available for review upon request.

## Abbreviations

ACTH: Adrenal cortical trophic hormone
BP: Blood pressure
CI: Confidence interval
CO: Cardiac output
eNOS: Endothelial nitric oxide synthase
ET-1: Endothelin-1
HR: Hazard ratio
NO: Nitric oxide
OS: overall survival
PFS: progression-free survival
PI: Proteasome inhibitor
RR: Relative risks
SVR: Systematic resistance
TKI: Tyrosine kinase inhibitor
VEGF: Vascular endothelial growth factor
VEGFR: VEGF receptor
